# Association of IFN-γ gene polymorphism and levels in HBV related disease chronicity in India

**DOI:** 10.1186/1471-2334-12-S1-P27

**Published:** 2012-05-04

**Authors:** Roli Saxena, YK Chawla, Jyotdeep Kaur

**Affiliations:** 1 Department of Biochemistry, Postgraduate Institute of Medical Education and Research, Chandigarh- 160 012, India; 2 Department of Hepatology, Postgraduate Institute of Medical Education and Research, Chandigarh- 160 012, India

## Background

Hepatitis B Virus (HBV) infection is a primary causative factor for hepatocellular carcinoma (HCC), the fifth most frequent cancer, worldwide. The present study evaluated the association of IFN-γ +874T>A polymorphism and its levels with HBV related HCC risk in Indian population.

## Methods

Five groups of subjects were enrolled viz. control (n=146), HBV-carriers (n=68), chronic active HBV (n=64), HBV-cirrhotics (n=60) and HBV-related HCC (n=59). Allele-specific-PCR was performed to study various polymorphic forms of IFN-γ (+874) and blood levels were estimated by ELISA. Genotype distribution was compared using chi square analysis and the odds ratios (ORs) and 95% CI were calculated to express the relative risk.

## Results

In IFN-γ (+874), the (TA) heterozygous genotype was found to be a significant risk factor for chronic-active hepatitis (OR=2.94, p<0.01) and HCC (OR=2.7, p<0.01) development, among controls and carrier groups, respectively. Similarly, the variant (AA) genotype, was also found to be significantly in positive association with HCC risk (p<0.01), among controls. Further, the spontaneous median IFN-γ levels were 151.09pg/mL in control population. While, the levels were significantly elevated (p<0.01) in HCC group (252.34pg/mL) in comparison to controls as well as other categories studied. However, no significant association was found between the genotype and the cytokine levels.

## Conclusions

IFN-γ polymorphic forms and its levels share a strong association with HBV-HCC risk in Indian population and thus, should be further evaluated as a candidate gene to determine individual susceptibility for the same.

